# Prognostic Significance of Neutrophil/Lymphocytes Ratio (NLR) in Predicting Recurrence of Cervical Dysplasia

**DOI:** 10.1155/2022/1149789

**Published:** 2022-04-11

**Authors:** Massimo Origoni, Francesco Cantatore, Giorgio Candotti, Massimo Candiani

**Affiliations:** Department of Gynecology & Obstetrics, Vita Salute San Raffaele University School of Medicine and IRCCS Ospedale San Raffaele Scientific Institute, Milan, Italy

## Abstract

**Objective:**

The objective of the present study was to investigate the potential prognostic role of neutrophil-lymphocyte ratio (NLR) in comparison with known parameters of prediction for the detection of recurrences of cervical intraepithelial neoplasia (CIN) after treatment.

**Methods:**

We retrospectively evaluated patients who underwent surgical treatment for CIN2, CIN3, and carcinoma in situ (CIN2+) between 2010 and 2019. NLR was recorded before surgery, and the follow-up records of patients were analyzed. Cases were splitted into two subgroups according to baseline NLR—low-NLR for <2 and high-NLR for ≥2 values of the index—and correlated with recurrences.

**Results:**

428 cases fulfilled the criteria and were included in the study. Recurrence rate in patients with NLR <2.0 and NLR ≥2.0 was 15.2% and 27.3%, respectively, being the odd ratio for recurrence significantly higher in patients with NLR ≥2 (OR = 2.09; 95% CI 1.28-3.41; *p* = 0.003). A highly significant statistical difference in recurrence rate was demonstrated, in both univariate and multivariate, for surgical margins, follow-up HPV-DNA status, and NLR values.

**Conclusion:**

Preoperative NLR categorization is a strong independent prognostic factor for recurrences after surgical excision of CIN. NLR evaluation is a simple, reproducible, and cost-effective clinical instrument that could optimally be introduced in clinical practice in every setting.

## 1. Introduction

The cervical intraepithelial neoplasia (CIN) represents the major risk factor for the development of invasive cancer and is almost exclusively due to high-risk human papilloma viruses (hrHPV) persistent infection [1]. The detection of histologically proven CIN2+ in cervical cancer screening programs, with few exceptions, represents an indication for surgical removal of the affected tissue in almost all recent international guidelines for cervical cancer prevention. In this view, the cervical conization represents the mainstay of treatment for intraepithelial lesions worldwide [2]. The patients conservatively treated for CIN2+ still remain at significant higher risk for cervical cancer development for up to 20 years [3], and overall recurrence rates of CIN after conization are reported to be as high as 20% during follow-up [4, 5]. It is a consistent report that the large majority of CIN recurrences are diagnosed in the first 24 months after treatment [4, 5]. Several factors are demonstrated being responsible for recurring CIN: age at diagnosis, HPV genotype, size and grade of the lesion, surgical margins of the cone involvement, and hrHPV persistence [6–8]. The association between inflammation and development of cancer has been well recognized [9, 10]. Within tumor microenvironment, chronic inflammation contributes to promote tumor growth, tumor angiogenesis, adjacent tissues invasion, metastatization, subversion of adaptive immune response, and reduced response to anticancer agents including hormones and chemotherapeutic agents [11, 12]. Among pretreatment parameters, one of the most recent application in clinical practice is represented by the peripheral neutrophil-to-lymphocyte ratio (NLR), which is regarded as a simple and effective marker of inflammation that has been reported to have an independent prognostic value in different tumors [13, 14]. An increased NLR index has been correlated with advanced stages of cervical cancer [15, 16], and the patients with more severe histologic grades of CIN have showed higher levels of total leukocytes [16], compared to low-grade CIN. The prognostic value of the NLR for predicting CIN recurrence after conization has also been recently investigated and reported in two studies from Iran and Korea [17, 18]. The objective of the present study is to retrospectively investigate the potential prognostic role of baseline neutrophil-to-lymphocyte ratio (NLR) in comparison with already known parameters of prediction for the detection of recurrence of CIN after treatment; the relative role of each parameter and the knowledge of the correlated relative risk would add benefit to the identification of at-risk subgroups of patients and would allow for potentially tailored, risk-based, follow-up strategies.

## 2. Materials and Methods

**Figure 1:**
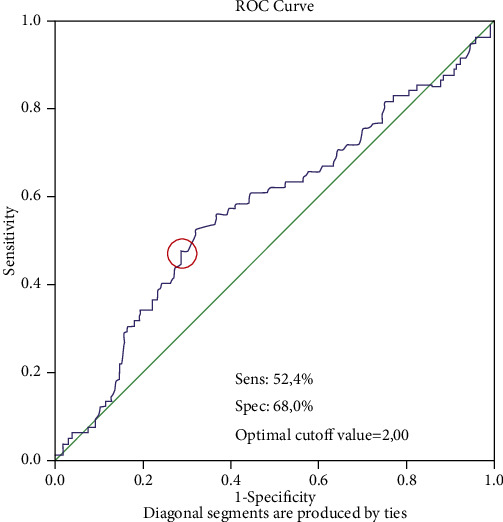
NLR receiver operating characteristic (ROC) curve analysis.

Records of all patients treated for high-grade cervical intraepithelial neoplasia (HG-CIN) between January 2010 and April 2019 at San Raffaele Hospital of Milan, Italy, were retrospectively considered and investigated. Cases included excisional cervical conservative treatments (cervical conization or loop electrosurgical excision procedure (LEEP)) performed for histologically detected, within the Italian cervical cancer screening program, CIN2, CIN3, and carcinoma in situ (CIN2+). The patients' characteristics including demographic data, histology, surgical and clinicopathologic variables, HPV-DNA status, and follow-up data at June 2020 were collected. The patients who underwent treatments for CIN recurrences, under immunosuppressive drugs regimens (e.g., organ transplant cases) or affected by immune system impairments (e.g., HIV positive cases), were excluded from the study. Excisional procedures were in all cases performed by trained colposcopists; preoperative, and follow up cytological and histological analysis were all performed by trained pathologists of the same internal staff. CIN recurrence was defined as CIN1+ pathological (cervical biopsy) detection during follow-up. According to our department policy, cervical conization is performed as a one-day surgery procedure, and all cases were preoperatively evaluated with blood tests including a complete blood count (CBC); thus, the baseline neutrophil/lymphocyte ratio index (NLR) was recorded and investigated. NLR data from the whole study group were utilized by generating a receiver operating characteristic (ROC) curve analysis and determining the Youden index [19]; an optimal cutoff value of 2.0 for predicting recurrences was identified for NLR index. Sensitivity (SE) and specificity (SP) of the NLR cutoff point of 2.0 for the diagnosis of CIN2+ recurrence were 0.524 and 0.680, respectively (Figure 1). On this basis, cases were splitted into two subgroups according to baseline NLR—low-NLR for <2 and high-NLR for ≥2 values of the index—and correlated with recurrences. The descriptive statistical analysis was performed to characterize the study population; in both groups, demographic, clinicopathological, and follow-up data were analyzed by the use of a Cox's regression model in univariate and multivariate fashion in relation to recurrence detection and prognostic significance. The multivariate analysis was performed including the independent variables that showed statistically significance at the univariate analysis. The statistical analysis was performed using the Statistical Package version 20.0 for Windows (SPSS). All *p* values are two-sided, and the significance was established for *p* < 0.05. In accordance with guidelines, the internal Institutional Review Board (IRB) approval was obtained after submission to Ethics Committee evaluation (protocol n. 84/INT/2020).

## 3. Results

**Figure 2:**
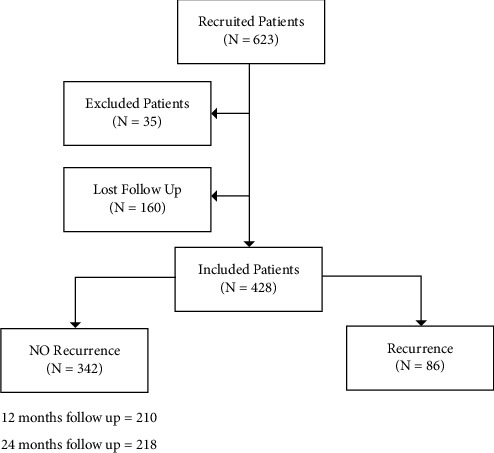
Study design.

Out of the whole cohort of 623 patients who were treated during the interval analysis and were considered as eligible, 428 cases fulfilled inclusion criteria and were included in the study, while 195 were excluded (160 being incompletely followed up according to study parameters and 35 lacking of complete clinical records) (Figure 2). Mean and median age of patients were 38.4 and 37.5 years, respectively; as far as it regarded the conization histology, a high-grade squamous intraepithelial neoplasia (HG-SIL) (CIN2, CIN3, and carcinoma in situ) was recorded in 90.4% of cases, while a low-grade squamous intraepithelial neoplasia (LG-CIN) (CIN1) was recorded in 9.1% of cases, and a high-grade glandular Intraepithelial neoplasia (HG-GCIN) (GCIN2 and GCIN3) was detected in 0.4% of the whole group. Surgical cone margins were positive in 15.2% of cases and negative in 84.8%. A follow-up human papilloma virus DNA testing was positive in 27% of cases and negative in 73%. The overall recurrence rate was 20.0% (18.2% within 12 months from conization and 1.2% within 24 months). The study cohort demographics and clinicopathological characteristics are summarized in Table 1. The NLR value was lower than 2.0 in 256 (59.8%) patients and was higher or equal to 2.0 in 172 (40.2%) patients. The cone histology, the surgical margins status, and the follow-up HPV DNA testing did not show significant differences between the two groups of NLR. The recurrence rate in patients with NLR <2.0 and NLR ≥2.0 was 15.2% and 27.3%, respectively, being the odd ratio for recurrence significantly higher in patients with NLR ≥2 (OR = 2.09; 95% CI 1.28-3.41; *p* = 0.003). When only HG-CIN (CIN2+) recurrences were considered for the same analysis, according to the baseline NLR value, a rate of 6.5% vs. 10.5% was recorded in the NLR<2 and NLR ≥2 group of patients, respectively (*p* = 0.05). Moreover, also in cases with negative surgical cone margins, the difference in terms of recurrences correlated with the NLR baseline values and revealed a significant higher rate of recurrences in the NLR ≥2 group compared to the NLR <2 group (23% vs. 12.4%, respectively; *p* = 0.008) (Table 2). When the recurrence risk assessment was performed by the use of univariate analysis considering age of patients, smoking, hormonal contraceptive use, surgical cone margins, follow-up HPV-DNA testing, and NLR values, a highly significant statistical difference in recurrence rate was demonstrated for surgical margins, follow-up HPV-DNA status, NLR values, and hormonal contraceptive use. At the multivariate analysis, the surgical margins, follow up HPV-DNA testing and NLR values were independently associated to recurrence; an oral contraceptive use was not significantly associated with recurrence.

The 95% CI relative odd ratios for recurrence were 3.53 for positive surgical cone margins, 3.00 for positive follow-up HPV-DNA testing, and 2.41 for NLR values ≥2, respectively (Table 3).

## 4. Discussion

The high-grade cervical intraepithelial neoplasia (HG-CIN) is characterized by a relatively high potential for recurrences after conservative surgery, being the great majority of these occurring in the first 2 years after treatment but with a significant prevalence up to ten years [3, 6, 7]. Different factors have been extensively demonstrated to be related to the recurrence rate: age of patients at diagnosis, surgical cone margins, and HPV positivity at follow-up [3, 6, 7, 20]; thus, the topic of predicting or, at least, identifying validated risk factors for recurrences is extremely relevant, both for the clinical and the investigational perspective. In this view, leukocytosis and neutrophilia are among the most frequently encountered alterations in cancer patients, and these findings significantly correlate with advanced disease and, consequently, with prognosis [14, 15, 16]. The neutrophil lymphocytes ratio (NLR) has been regarded as a noninvasive and cost-effective marker that reflects systemic inflammatory conditions, and many studies have reported its valuable prognostic correlation in different cancers. Studies on several solid cancers such as colorectal cancer, squamous cell carcinoma of the lung, and thymoma revealed that high NLR levels prior to surgery were significantly associated with worse survival, and the patients with a higher NLR level had a shorter disease free survival (DFS) [21–23]. The applicability of NLR has recently been preliminarily demonstrated also in neoplastic diseases having the same HPV etiology as cervical cancer, developing in the oral cavity [24]. Moreover, there is strong evidence that laboratory biomarkers easily obtained in routine clinical practice can have role in outcome prediction in a variety of medical conditions, not limited to cancers, including major cardiac events [25], cerebral hemorrhage [26], and ischemic stroke [27, 28].

The major relevance of the present study is the demonstration that the recurrence of preneoplastic lesions of the cervix, as already shown for other tumors, can also be predicted with the use of NLR. Uterine cervical cancer is one of the most common malignancies and leading cause of cancer death among women worldwide, and it is preceded by a pre-invasive step in which defective, impaired neutrophil migration could be an early event in tumor development. To our knowledge, the current series is the first study conducted in Europe and represents the largest series available, taking into consideration NLR as a prognostic factor for predicting the likelihood of recurrence in patients who underwent excisional conservative procedures for CIN. Only two previously published studies investigated the relationship between NLR and the recurrence of CIN in 230 and 307 patients, respectively [17, 18]. The patients were divided into two groups, low and high NLR groups, and they were, in both studies, demonstrated having different recurrence rates of CIN according to the preoperative NLR values. The same results have been obtained in our studied cohort of patients. Of note, in our study, we exclusively enrolled patients with high-grade CIN (CIN2/3), as it is currently accepted worldwide that only high-grade CIN is significantly associated with cancer risk, while low-grade CIN (CIN1) has no cancer potential. Chun et al. and Farzaneh et al., in those two previous studies [17, 18], also enrolled patients with CIN1 in 6.5% and 15% of their population, respectively. This factor could definitely better explain the recurrence rate in our series, which is very likely to correlate with the real risk of recurrence in these preneoplastic conditions. It is well known that high-risk HPV (hrHPV) persistence after treatment is correlated with high risk of recurrence after conization for CIN and there is a general consensus in literature that hrHPV positive testing during a clinical follow-up of patients after treatment for CIN is one of the major negative prognostic factors for recurrence [1, 3, 6, 8]. For this reason, also in our study, all selected patients were tested for hrHPV after conization, and 30% of them tested positive for hrHPV infection after treatment. A univariate and multivariate analysis confirmed post-treatment hrHPV persistence as an independent prognostic factor for recurrence. Accordingly with published and well-accepted results of several studies, we found a strong correlation with diseases recurrence in patients with positive surgical margins at the conization surgical specimen. This finding is consistent with the results of several authors [4–7, 20] and the evidences of many international guidelines on CIN recurrence-associated risk factors [29]. In this view, some authors have proposed reconization in case of margins involvement; however, this strategy is still controversial, mainly due to the negative effects on subsequent pregnancies of repeated excisional procedures on the cervix [30, 31]. In this particular setting of patients, together with an overall clinical assessment, NLR evaluation could positively be of help in the decision-making process of identifying an objective risk factor for recurrence and thus the decision of a retreatment vs. an observational follow-up strategy. In fact, preoperative NLR values could act as an effective prognostic biomarker especially in young patients, in which the potential negative impact of a second cervical treatment on subsequent fertility and pregnancy is particularly relevant; moreover, the identification of patients with a higher likelihood of recurrence will allow a personalized follow-up protocols splitting between high-risk and low-risk individuals. In our series of patients, NLR has been shown to be a robust significant prognostic factor even considering high-grade recurrences only, excluding recurring low-grade lesions; in our opinion, this result is particular relevant as it precisely correlates with the clinical need of correctly identifying high-risk cases and correctly managing their postoperative management, avoiding intensive diagnostic procedures in low-risk cases. Moreover, a significant prognostic value of the NLR has been also demonstrated when cases with positive surgical margins at conization were excluded from the analysis; this, in our opinion, provides even more strength to our findings. In our study, despite the interesting results, some limitations must be taken into consideration and underlined. First, the retrospective nature of the study might be seen as a kind of bias; nonetheless, the largest majority of studies focusing on the prevalence of recurrences after conization relies on retrospective analysis and data interpretation. We firmly believe that our results support the option of including the preoperative NLR categorization in the parameters to be evaluated in prospective randomized trials. Second, the follow-up interval analyzed in our experience for the detection of recurrences, established in 24 months, does not completely reflect the overall prevalence of recurrences, as it has been demonstrated that these patients are at higher risk up to 10 years after treatment [3, 32], compared to the general population. Despite this, it has also been reported that almost 90% of the recurrences occurs within the first 2 years after treatment [4, 5], thus allowing an acceptable data interpretation.

We believe that the strength and the validity of the study rely on the following features: Our experience reports the largest cohort of patients analyzed worldwide, with stringent parameters of considering clinically relevant recurring lesion only (high-grade CIN) by the use of a simple, reproducible, and cost-effective clinical instrument that could optimally be introduced in clinical practice in every setting. Prospectively, it could be interesting and useful also to investigate, within the histopathological work up of the cervical cone, the features and the degree of inflammatory cells infiltration around the lesion, with the aim of identifying a similar pattern of NLR values as it has been done in serum samples; this could potentially add further information concerning the natural history and the risk factors for recurrences after conization. Moreover, this parameters could optimally and positively been adopted in early-stage cervical cancer, for which conservative treatments in young patients are becoming the firstline option in many instances. In conclusion, we demonstrated that preoperative NLR categorization is a strong independent prognostic factor for recurrences after surgical excision of CIN. This simple parameter might provide additional prognostic value beyond what conventionally obtained by clinical-pathological and biomolecular parameters.

## Figures and Tables

**Table 1:** Demographics and clinical-pathological parameters of study cohort.

Study cohort (*N* = 428)		
Age (years)		
Min.	18	
Max.	73	
Media	38.4 (±9.5)	
Median (I.Q.R.)	37.5 (31-44)	
Cone histology	CIN 1	39 (9.1%)
	CIN 2	192 (44.9%)
	CIN 3	162 (37.9%)
	CA “in situ”	33 (7.7%)
	G-CIN 2	1 (0.2%)
	G-CIN 3	1 (0.2%)
HPV post-treatment		
	Positive	115 (27%)
	Negative	313 (73%)
Margins		
	Positive	65 (15.2%)
	Negative	363 (84.8%)
Recurrences		
	Yes	86
		*At 12* months*: 78*
		*At 24* months*: 8*
	No	342

**Table 2:** NLR subgroups and risk factors analysis.

	NLR <2 (*N* = 256)	NLR ≥2 (*N* = 172)	*p*
Age (years)	38.0 (±9.7)	39.0 (±9.4)	0.484
WBC (mean)	6412.5 (±1523.8)	7801.1 (±1916.8)	<0.001
Lymphocyte	2371.1 (±569.2)	1913.9 (±496.3)	0.123
Neutrophil	3392.6 (±994.6)	5187.2 (±1504.7)	<0.001
Histology			
CIN 1	27 (10.5%)	12 (7.0%)	
CIN 2	114 (44.5%)	78 (45.3%)	
CIN 3	96 (37.5%)	66 (38.4%)	0.597
CA “in situ”	17 (6.6%)	16 (9.3%)	
G-CIN 2	1 (0.4%)	/	
G-CIN 3	1 (0.4%)	/	
Margins			
Positive	38 (14.9%)	25 (15.3%)	0.518
Negative	215 (85.1%)	138 (84.7%)	
			
HPV post-treatment			
Positive	76 (30%)	39 (24%)	0.166
Negative	180 (70%)	133 (76%)	
Recurrences	39/256 (15.2%)	47/172 (27.3%)	0.003
CIN2+ recurrences	17/256 (6.5%)	18/172 (10.5%)	0.05
Recurrences In free margins cones	27/218 (12.4%)	34/146 (23%)	0.008

**Table 3:** Univariate and multivariate recurrence risk assessment.

	Rec +	Rec -	Univariate	Multivariate
Age (years)				
≤30	23	73	*N.S.*	*—*
31-44	39	189		
≥45	24	80		
NLR				
<2	39	217	*p* = 0.002	*p* = 0.001
≥2	47	125	*OR (CI 95%):* 2.09 (1.30–3.37)	*OR (CI 95%):* 2.41 (1.44–4.02)
Hr-HPV (post-LEEP)				
Negative	50	263	*p* = 0.001	*p* < 0.001
Positive	36	79	*OR (CI 95%):* 2.40 (1.46–3.93)	*OR (CI 95%):* 3.00 (1.76–5.12)
Margins				
Positive	25	40	*p* < 0.001	*p* < 0.001
Negative	61	302	*OR (CI 95%):* 3.42 (1.80–5.64)	*OR (CI 95%):* 3.53 (1.91–6.52)
Smoking				
Yes	37	139	*N.S.*	*—*
No	49	203		
E/P				
Yes	15	97	*p* = 0.042	*p* = 0.096
No	71	245		

Rec = recurrence; N.S. = not significant.

## Data Availability

The data used to support the findings of this study are available from the corresponding author upon request.

## Abbreviations

NLR: Neutrophil-lymphocyte ratio
CIN: Cervical intraepithelial neoplasia
hr-HPV: High-risk human papilloma viruses
HG-CIN: High-grade cervical intraepithelial neoplasia
LG-CIN: Low-grade squamous intraepithelial neoplasia
LEEP: Loop electrosurgical excision procedure
CBC: Complete blood count
ROC: Receiver operating characteristic
SE: Sensitivity
SP: Specificity
IRB: Institutional review board
DFS: Disease free survival.
